# Volume Change Measurements of the Heart and Lungs After Pectus Excavatum Repair

**DOI:** 10.3390/jcm14124250

**Published:** 2025-06-15

**Authors:** Gongmin Rim, Kwanyong Hyun, Hyung Joo Park

**Affiliations:** 1 Department of Thoracic and Cardiovascular Surgery, Cha Medical University Bundang Cha Hospital, Seongnam 13497, Republic of Korea; yim8585@chamc.co.kr; 2 Department of Thoracic and Cardiovascular Surgery, The Catholic University of Korea St. Vincent’s Hospital, Suwon 16247, Republic of Korea; pipedragon@gmail.com; 3 Department of Pediatric & Thoracic Surgery, Cleveland Clinic Pectus and Chest Wall Institute, Cleveland Clinic Children’s Hospital, 8950 Euclid Ave/R3, Cleveland, OH 44106, USA

**Keywords:** Pectus excavatum, XI technique, volumetric measurement, cardiac volume

## Abstract

**Background/Objectives:** The primary objective of PE repair is to relieve compression exerted on the cardiac and pulmonary structures and enhance the thoracic cavity volume. However, the number of volumetric studies of the thoracic cavity, including the heart and lung volumes, is scarce. This study seeks to systematically evaluate the volumetric changes in these structures to assess the physiological impact obtained by PE repair. **Methods:** A retrospective analysis was conducted on 63 patients who underwent PE repair using the XI bar technique from April 2023 to February 2024. Volumetric changes were measured preoperatively and postoperatively using SYNAPSE 3D imaging software (Version 4.6, Fujifilm, Tokyo, Japan). Cardiac and pulmonary volumes were quantified, and CT indexes (Haller index, Depression index) were assessed. Complication rates, reoperation rates, and length of hospital stay were also analyzed. **Results:** The mean cardiac volume increased significantly from 458.25 mL preoperatively to 499.13 mL postoperatively (*p* = 0.018), showing an 8.9% increase. Pulmonary volumes, however, showed no statistically significant change, remaining stable at approximately 4371.31 mL preoperatively and 4266.87 mL postoperatively (*p* = 0.57). **Conclusions:** Repairing PE markedly enhances cardiac volume, emphasizing its importance in relieving mediastinal compression. Pulmonary volumes remain largely unaffected, suggesting that PE primarily impacts cardiac structures. Our approach to the volumetric measurements provides valuable insights into the physiological outcomes of chest wall remodeling and is considered to be a good modality for future studies to enhance our understanding of the functional benefits of PE repair.

## 1. Introduction

Pectus excavatum (PE), the most prevalent congenital chest wall deformity, is characterized by a sunken sternum, resulting in a concave chest wall configuration [1,2,3]. Over the past two decades, advancements in minimally invasive surgical techniques and instrumentation for PE repair have led to significant improvements in patient outcomes [4,5,6,7]. Despite these advancements, the extent to which these procedures enhance physiological capacity, particularly in terms of increased cardiac and pulmonary volumes, remains insufficiently studied. It is not clearly understood how these procedures enhance physiological capacity in terms of cardiac and pulmonary volumes. Traditional assessment methods, such as the Haller index (HI) and Depression index (DI), primarily focus on the anatomical severity based on specific CT measurements. While these indices provide a general understanding of chest morphology, they fall short of directly evaluating the physiological impact of PE repair, particularly the cardiac and pulmonary functions. Additionally, these indices are prone to variability due to differences in imaging protocols and operator interpretation. To overcome these limitations, advanced volumetric imaging techniques have emerged as a valuable alternative. By enabling comprehensive, three-dimensional assessments of cardiac and pulmonary volumes, tools like the SYNAPSE 3D imaging software (Version 4.6, FUJIFILM Corporation, Minato, Tokyo, Japan) provide precise and reliable data on the physiological outcomes of PE repair. SYNAPSE 3D imaging software was developed as a method enabling comprehensive three-dimensional volume measurement. We adapted this algorithm to assess the intrathoracic organ volume changes to look for the physiological benefits from PE repair. This study aims to measure cardiac and pulmonary volumes and integrate them into the evaluation of our comprehensive chest wall remodeling approach using the XI pectus bar technique, assessing its impact on these volumes.

## 2. Materials and Methods

### 2.1. Study Design

This study was approved by the Institutional Review Board (NR-IRB 2024-02, approved on 15 April 2024), with a waiver of informed consent granted due to its retrospective design. A retrospective review was conducted on 63 out of 74 patients who underwent PE repair surgery using XI pectus bars between April 2023 and February 2024 at a single center. Exclusion criteria included (1) patients with a history of prior PE repair resulting in recurrence, (2) patients who underwent alternative pectus bar configurations, such as parallel or cross-bar techniques, and (3) patients with incomplete medical records. All surgeries were performed by the corresponding author (H.J.P). Data collected included demographic information, medications administered, surgical and medical histories, and perioperative data (e.g., operative time, complications, length of hospital stay [LOS], and chest computed tomography [CT] findings). This information was obtained through patient interviews and electronic medical records.

### 2.2. Surgical Procedures

**Figure 1:**
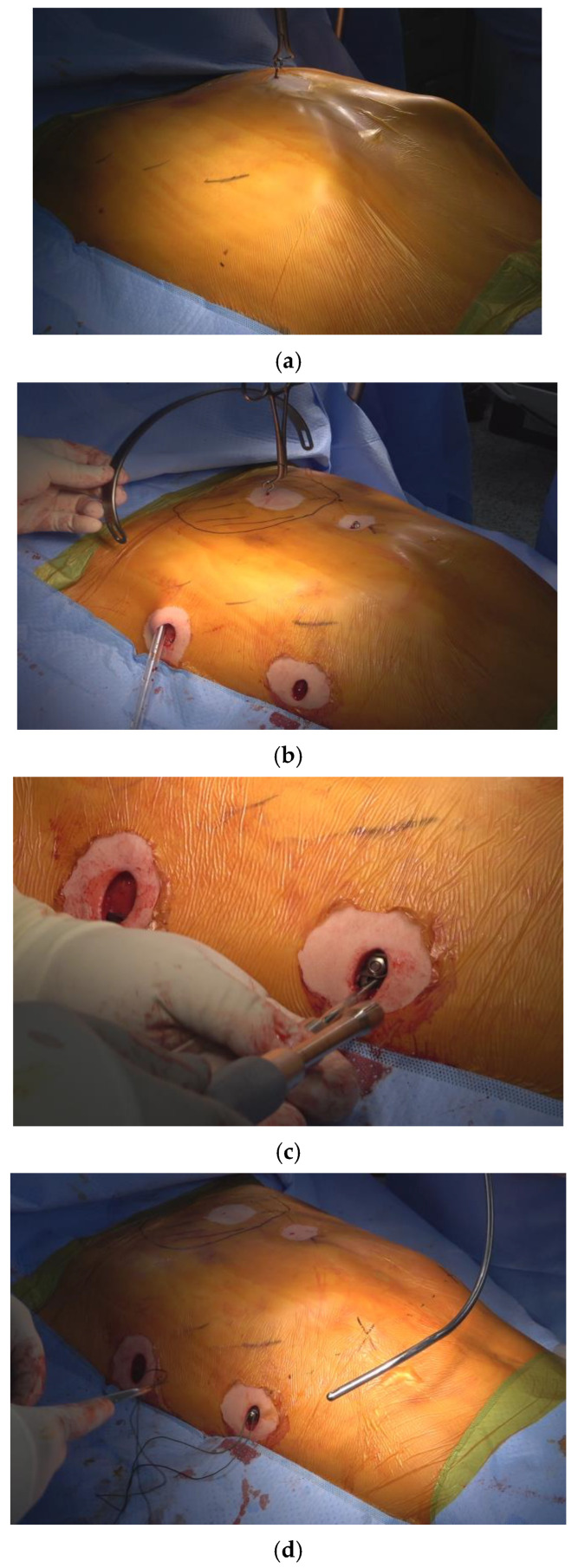
The four steps involved in crane-powered entire chest wall remodeling were as follows: (**a**) Total elevation of the sternum using a screw crane to exceed the desired chest wall remodeling level. (**b**) Introduction of multiple pectus bars at the bilateral anterior axillary lines in an XI configuration for comprehensive chest wall remodeling. (**c**) Stabilization of pectus bars with bilateral bridge plates, linking the bars using nuts and bolts at the subcutaneous plane. (**d**) Correction of lower costal flares and residual protrusions using the Flare-buster and Magic string technique with heavy strings.

All the repair procedures were performed by a single surgeon at a single center, ensuring consistency in surgical technique, except for the nerve block procedure. Patients were positioned supine with their arms suspended in overhead sling. Bilateral 1.5 cm skin incisions were made along the midaxillary line to create subcutaneous pockets. The operative technique for comprehensive chest wall remodeling consisted of four key steps (Figure 1) [4]:

*Four Essential Steps in Pectus Excavatum Repair using the Crane-Powered Entire Chest Wall Remodeling Strategy*

1. Crane-assisted sternal pre-lifting: The sternum was elevated beyond its normal anatomical level using a crane system (Easy Crane®, PrimeMed Co., Ltd., Seongnam-si, Gyeonggi-do, South Korea). in all cases, regardless of patient age or deformity severity. This technique expanded the mediastinal space, which is typically tightly compressed between the heart and chest wall, thereby allowing safe retrosternal dissection. By removing the burden of lifting from the pectus bar, it also enabled precise bar placement and minimized rotational force, reducing the risk of injury to adjacent structures [8,9,10].

2. Introduction of multiple pectus bars in an XI configuration: Three pectus bars were inserted through bilateral mid-axillary incisions and arranged in an XI pattern—with two lower cross bars positioned below the nipple line and one upper horizontal bar above it. This configuration maximizes anterior chest wall coverage. Since 2022, the XI bar strategy has been our standard approach for comprehensive chest wall remodeling [3,4].

3. Pectus bar stabilization using bilateral bridge plates: The bars were rigidly fixed by connecting them with bilateral subcutaneous bridge plates secured with bolts and nuts. This method effectively prevents bar displacement and eliminates the need for conventional stabilizers, which have shown limited effectiveness [6,11].

4. Chest wall ironing using the Flarebuster and Magic String techniques: To achieve full anterior chest wall remodeling, lower costal flares and focal protrusions were compressed using a modified subcutaneous sandwich technique. This involved No. 5 Ethibond^®^ sutures (Ethicon Inc., Somerville, NJ, USA) to securely tether the chest wall, helping to restore a more natural contour [4,12,13].

### 2.3. Three-Dimensional Synapse Program and Volumetric Measurement

For volumetric measurements, SYNAPSE 3D imaging software (Fujifilm, Tokyo, Japan) was utilized. This program has been validated in prior studies for its ability to provide high-resolution, automated segmentation of anatomical structures from CT images [14,15].

The following steps outline the measurement process in detail:

(1) CT data acquisition: Data for volume measurements were obtained from preoperative and postoperative chest CT scans. Preoperative scans were conducted as part of the routine evaluation to establish baseline anatomical and volumetric parameters. Postoperative scans were acquired on the third day following surgery to accurately assess immediate volumetric changes while minimizing the influence of transient postoperative factors, such as edema or inflammation. All scans were performed with a 1 mm slice thickness to ensure high-resolution imaging for precision of volumetric analysis. Imaging was conducted using a multi-detector computed tomography system, ensuring uniformity and consistency across the patient cohort. Non-enhanced CT protocols were utilized to mitigate potential risks associated with contrast agents, particularly considering that the majority of patients were pediatric.

(2) Volumetric calculations: SYNAPSE 3D program calculates volume by automatically segmenting anatomical structures based on predefined density thresholds from CT images. The software employs an advanced algorithm that distinguishes cardiac and pulmonary structures by analyzing voxel intensity values and applying a region-growing technique. Following segmentation, a three-dimensional reconstruction of the selected region is generated, and volume is computed by integrating the voxel-based segmentation data. For cardiac volume analysis, the 4-chamber analysis mode was employed to segment the heart. This mode automatically distinguishes the myocardium, left and right atria, and ventricles. The program generates 3D renderings and multiplanar reconstructions, allowing for precise quantification of total cardiac volume. Manual adjustments were performed when necessary to ensure segmentation accuracy. And for pulmonary volume analysis, the lung analysis/airway mode was used to segment pulmonary structures. This mode isolates lung fields and lobes based on density thresholds, effectively differentiating air-filled spaces from surrounding tissues. The software provides both individual lobe volumes and total lung volume.

(3) Quality Control, Accuracy, and Reliability of Volumetric Measurements: To ensure accurate volumetric assessments, all automated segmentations were independently reviewed by two thoracic surgeons. Discrepancies between automated results and manual evaluations were resolved through consensus, and volume measurements were cross-referenced with anatomical landmarks to validate segmentation reliability. SYNAPSE 3D has been previously validated in comparative studies against manual segmentation and other imaging software, demonstrating high inter-observer and intra-observer reliability [14,15]. The accuracy of volumetric measurements was further enhanced through manual verification and correction by independent experts, ensuring precise delineation of organ boundaries. This automated yet expert-supervised approach facilitates reproducible and objective assessments of postoperative volumetric changes, minimizing interobserver variability and improving the robustness of our study.

### 2.4. Statistical Analysis

Normally distributed continuous data, skewed data, and categorical data are presented as means (standard deviation [SD]) values, medians (interquartile ranges), and numbers, respectively. Descriptive statistics (number of individuals, mean, SD, median, minimum, maximum, and quartile range) and categorical data were conveyed as subject numbers and percentages, checked for normal distribution and relations, and assessed using analysis of variance or the Kruskal–Wallis test. All computations were performed using standard software (SPSS v. 25.0; IBM Corp., Armonk, NY, USA).

## 3. Results

A total of 63 patients were enrolled in this study. The baseline characteristics of the study cohort are detailed in Table 1. The mean age of the patients was 16.95 years (range: 11–37 years), with a predominance of male patients (52, 82.54%). The majority of the patients were Asian (96.82%), while the remainder were Caucasian (3.18%). Perioperative characteristics are summarized in Table 2. The chest morphology was symmetric in 40 patients (63.5%) and asymmetric in 23 patients (36.5%).

All patients underwent sternal pre-lifting using the crane, introduction of XI-shaped customized pectus bars, stabilization of the bars with bilateral bridge plates, and chest wall ironing using the flare buster and magic string technique. The mean operative time was 98.57 min (range: 65–150 min), and there were no intraoperative blood transfusions. Radiological indices, including the HI and DI, significantly decreased postoperatively. The mean difference in the HI was 1.65 (range: 0.53–4.3, *p* < 0.01), and the mean difference in the DI was 0.85 (range: 0.3–2.23, *p* < 0.01). Postoperative outcomes, including length of stay and complications, are summarized in Table 3. The mean length of stay was 4.52 days (range: 4–7 days). There were no severe complications such as major bleeding or bar displacement. Postoperative pneumothorax occurred in eight patients (12.7%), which required no intervention other than the connection of previously inserted small-bore silastic drainage to the digital thoracic drainage system (Thopaz^®^, Medela, Switzerland). One patient developed a hemothorax, requiring chest tube drainage; two patients had pneumonia; one patient developed a wound seroma; and one patient experienced thoracic outlet syndrome, which resolved spontaneously.

**Figure 2:**
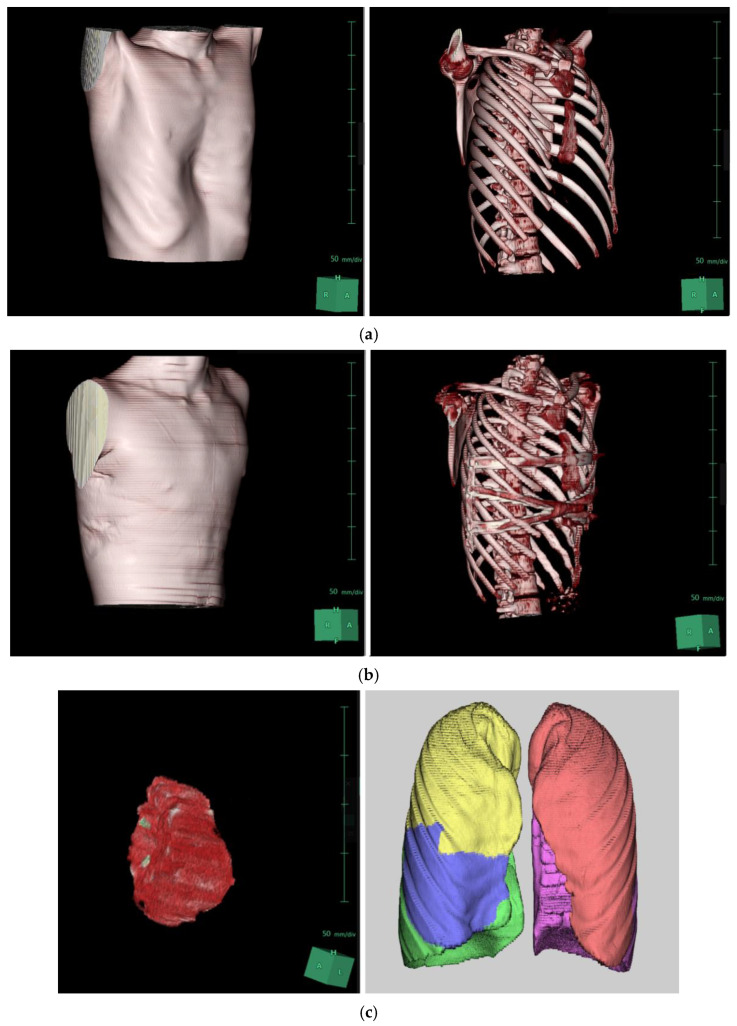

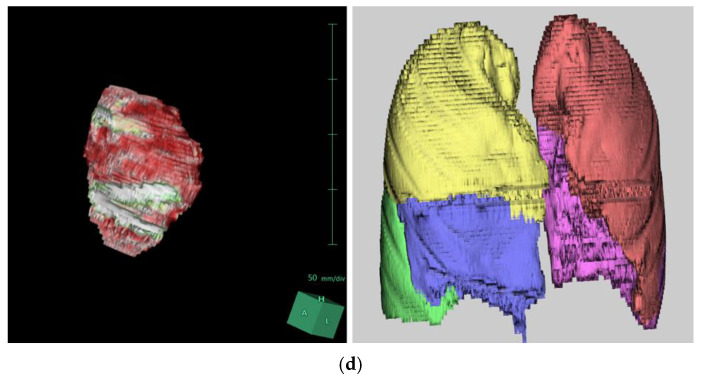
Three-dimensional reconstruction of the patient’s chest CT scan using SYNAPSE 3D: (**a**) Preoperative images. (**b**) Postoperative images following XI repair. (**c**) Three-dimensional reconstructed images of the heart and lungs before the repair. (**d**) Three-dimensional reconstructed images of the heart and lungs after the repair.

Chest CT scans were analyzed using the Synapse 3D image analysis program, focusing on heart and lung volumes, with results summarized in Table 4. There were no significant changes in lung volumes (total, left, and right) postoperatively. However, cardiac volumes increased from 458.25 mL to 499.13 mL after surgery (*p* = 0.018), representing an 8.9% increase with a mean difference of 40.89 mL (Figure 2).

## 4. Discussion

**Figure 3:**
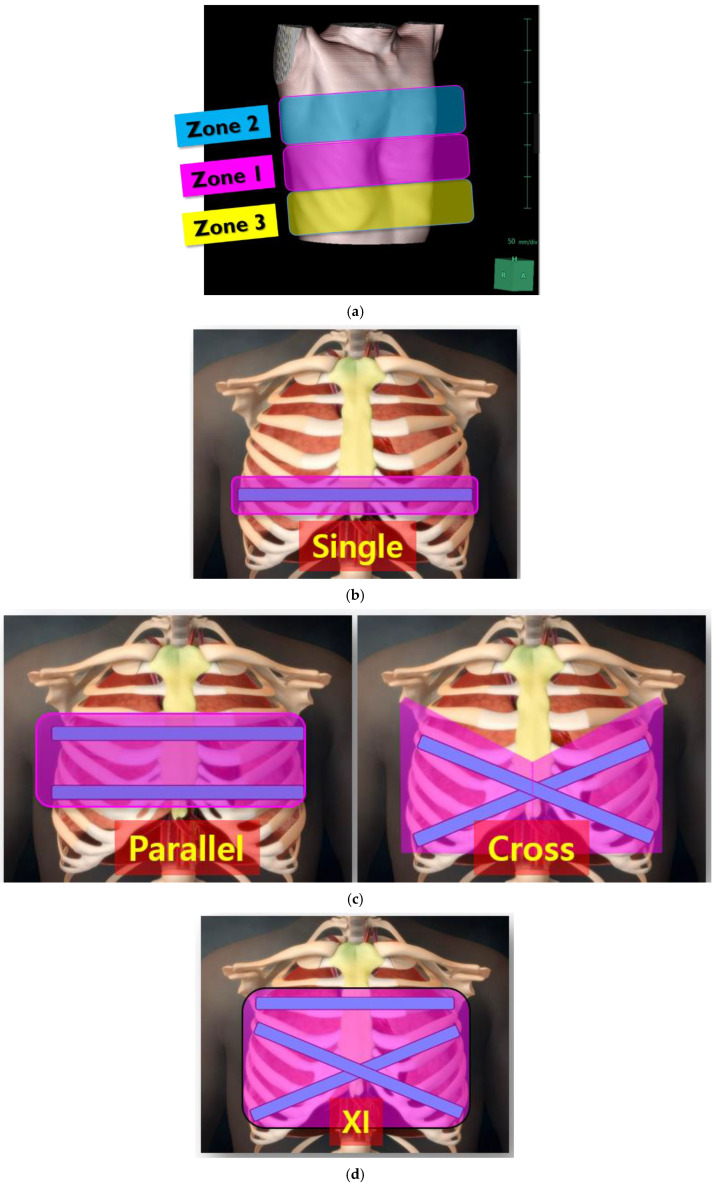
Three zones of chest wall deformities and coverage areas of different pectus bar configurations. (**a**) Zone 1: deepest point of the sternum; Zone 2: upper chest wall deformity; Zone 3: lateral chest wall and lower costal flares. (**b**) Coverage area of a single pectus bar, primarily addressing Zone 1. (**c**) Coverage area of double pectus bars: parallel-shaped bars addressing Zones 1 and 2, and cross-shaped bars covering Zones 1 and 3. (**d**) Coverage area of the XI pectus bar, comprehensively addressing Zones 1, 2, and 3.

PE repair aims to correct chest wall deformities and mitigate the compression of underlying structures, particularly the heart and lungs. We have been adopting a comprehensive chest wall remodeling approach to restore the thoracic cage to its integral anatomical structure and normal volume. To optimize repair outcomes, our center has categorized chest wall deformities into three zones to guide targeted surgical strategies (Figure 3a). Zone 1 represents the deepest point of the sternum and is the primary focus of repair. Zone 2 encompasses upper chest wall deformities, while Zone 3 includes the lower costal flares and lateral chest wall. Traditional single-bar techniques, such as the Nuss procedure, primarily address Zone 1 (Figure 3b). Double-bar configurations, including parallel and cross-shaped designs, extend coverage to Zones 1 and 2 or Zones 1 and 3, respectively (Figure 3c). However, these approaches often leave parts of the chest wall inadequately corrected. The XI bar configuration, representing an evolution in PE repair techniques, has emerged as a comprehensive solution capable of addressing all three zones (Figure 3d).

By providing robust stabilization and complete remodeling of the anterior chest wall, this technique minimizes the risks of bar dislocation and incomplete correction which are associated with traditional approaches. Our findings suggest that the XI bar technique delivers consistent outcomes with lower complication rates and greater patient satisfaction, aligning with recent advancements in chest wall repair.

Volumetric analysis offers significant advantages over traditional metrics such as the HI and DI in evaluating the physiological benefits of PE repair. While HI and DI effectively quantify morphological severity, they do not capture direct changes in cardiac and pulmonary volumes, limiting their capacity to assess functional outcomes. Previous studies have highlighted the impact of PE on cardiopulmonary function and the improvements observed post-repair. For instance, Jaroszewski et al. reported notable enhancements in cardiopulmonary performance among adult patients undergoing PE surgery, including increases in right ventricular stroke volume and cardiopulmonary exercise test parameters [16]. Systematic reviews further corroborate these findings, with one study documenting a 36% increase in cardiac output immediately following surgical correction [17]. The SYNAPSE 3D imaging software represents a precise, automated, and reproducible method for volumetric analysis, enabling comprehensive assessments of structural and functional changes post-repair. In this study, SYNAPSE 3D was employed to evaluate changes in cardiac and pulmonary volumes, providing critical insights into the physiological impact of the XI bar technique. Our findings demonstrated a significant 8.9% increase in cardiac volume (*p* = 0.018) after PE repair, reinforcing the procedure’s effectiveness in alleviating mediastinal compression. This result is consistent with prior research that reported improvements in cardiac function metrics, including stroke volume and ejection fraction, following surgical correction. The increase in cardiac volume observed in this study underscores the importance of addressing the mechanical impact of chest wall deformities.

Conversely, pulmonary volumes showed no significant changes post-repair, consistently with prior studies [18,19,20]. This stability suggests that lung parenchyma, due to its inherent compliance and adaptability, is less directly affected by external compression. While volumetric analysis indicates static pulmonary volumes, dynamic measures such as spirometry have demonstrated modest improvements in respiratory function post-repair. This disparity highlights the need for comprehensive assessments that integrate both static and dynamic pulmonary metrics to fully understand the impact of PE repair on lung function.

The cases analyzed involve the XI bar configuration because they represent our most recent cohort. This study was not designed to compare outcomes between single-bar, parallel-bar, or cross-bar techniques. Rather, our focus was on assessing the volumetric gain before and after repair. The goal was not to compare surgical techniques but to quantify the volume increase achieved with our method.

Our current pectus repair strategy emphasizes entire chest wall remodeling using multiple bars, rather than simply elevating the depression. The decision to use multiple bars is based on the extent of the chest wall deformity, not just the severity of the depression. Our primary aim is to restore the entire chest wall to a more normal configuration, which is why the XI bar technique has been used in all recent cases. Further details of this technique will be outlined in upcoming publications.

To evaluate the effectiveness among different bar configurations (e.g., single-bar or alternative multi-bar techniques), future studies could include volumetric analyses across defined chest wall zones (Zones 1–3).

To our knowledge, this is the first study to investigate volumetric changes in the heart and lungs following PE repair using the XI bar technique. While the findings provide valuable insights, the study has several limitations. First, the sample size was relatively small, which may limit the generalizability of the results. Second, the retrospective nature of the study introduces potential biases. Third, the use of non-enhanced CT scans restricted the ability to analyze volume changes in individual heart chambers or differentiate between cardiac regions. Fourth, artifacts from the pectus bars may have influenced volumetric calculations. Finally, the absence of complementary assessments, such as echocardiograms or cardiopulmonary exercise testing, prevented the establishment of correlations between volumetric changes and functional outcomes.

Future research should focus on addressing these limitations and expanding the scope of volumetric analysis to compare different pectus bar configurations. Longitudinal studies incorporating functional assessments and quality-of-life metrics will further elucidate the clinical relevance of volumetric changes and enhance our understanding of the long-term benefits of PE repair.

## 5. Conclusions

The repair of PE using the XI pectus bar technique demonstrates a significant increase in cardiac volume, highlighting its efficacy in relieving mediastinal compression. In contrast, pulmonary volumes remain largely unchanged, suggesting that the primary physiological impact of the repair is localized to cardiac structures. These findings underscore the critical role of volumetric assessments in advancing our understanding of the functional benefits associated with PE repair. Future research should aim to establish robust correlations between volumetric changes and long-term functional outcomes, including cardiopulmonary performance and quality-of-life improvements. Such studies will enhance the generalizability of these findings and provide deeper insights into the relationship between volumetric changes and functional metrics, ultimately guiding more comprehensive evaluations of surgical efficacy.

## Figures and Tables

**Table 1:** Baseline characteristics of the study group.

	XI Repair (*n* = 63)
Age, years (range)	16.95 (11–37)
Height, cm (range)	169.35 (146–185)
Weight, kg (range)	53.20 (32–87)
BMI, kg/m^2^ (range)	18.26 (14.2–25.1)
Male sex (percentage)	52 (82.54)
Race, Asian (percentage)	61 (96.82)
ASA class (percentage)	
ASA I	61 (96.82)
ASA II	2 (3.18)

ASA, The American Society of Anesthesiologists.

**Table 2:** Perioperative characteristics of the study group.

	XI Repair (*n* = 63)	*p* Value
**Depression index (DI)**		
Pre (range)	1.87 (1.39–3.3)	
Post (range)	1.02 (1.00–1.09)	
Δ DI	0.85 (0.3–2.23)	<0.01
**Haller index (HI)**		
Pre	4.07 (3.02–7.67)	
Post	2.38 (1.39–3.37)	
Δ HI	1.65 (0.53–4.3)	<0.01
**Morphological type**		
PE, symmetric type	40 (63.50)	
PE, asymmetric type	23 (36.50)	
Operative time, min	98.57 (65–150)	
Intraoperative transfusion	0 (0)	
Pectus bar size, inch	13.49 (11–15)	
**Pectus bar shape**		
XI	63 (100)	
**Number of pectus bars**		
3	63 (100)	
**Pectus bar stabilization**		
Bridge plate	63 (100)	
Crane application	63 (100)	
Pectoscope	63 (100)	
Flare buster	63 (100)	
Magic string	63 (100)	

*p* < 0.05 Data expressed as mean (range) or number (%) PE, Pectus excavatum.

**Table 3:** Postoperative outcomes in the study group.

	XI Repair (*n* = 63)
Length of stay, days	4.52 (4–7)
**Complications**	
Pneumothorax	8 (12.70)
Hemothorax	1 (1.6)
Pneumonia	2 (3.2)
Wound complications	1 (1.6)
Thoracic outlet syndrome (TOS)	1 (1.6)
Bar dislocation	0
Re-operation	0
Readmission in 30 days	0

*p* < 0.05 Data expressed as mean (range) or n (%).

**Table 4:** Volumetric changes after the XI bar repair.

	XI Repair (*n* = 63)	*p* Value
**Lung volume**		
**Total**		
Pre (range)	4371.31 (2034.1–6447.6)	
Post (range)	4266.87 (2385.3–6446.8)	
Δ volume	−104.42 (−569.6–712.3)	0.52
**Left**		
Pre (range)	2052.51 (929.5–3004.6)	
Post (range)	2022.61 (1111.1–3048.2)	
Δ volume	−29.90 (−713.2–347.3)	0.64
**Right**		
Pre (range)	2318.77 (1104.5–3443)	
Post (range)	2310.86 (1268.4–3418.6)	
Δ volume	−7.90 (443.2–395.6)	0.70
**Heart volume**		
Pre	458.25 (289.8–647.4)	
Post	499.13 (332.7–726.5)	
Δ volume	40.89 (−35–170.35)	0.018

*p* < 0.05 Data expressed as mean (range, SD) or n (%).

## Data Availability

The datasets used in the current study are available from the corresponding author on reasonable request.

## Abbreviation

Pectus excavatum (PE).
